# Microfibril-associated protein 5 and the regulation of skin scar formation

**DOI:** 10.1038/s41598-023-35558-x

**Published:** 2023-05-30

**Authors:** Chen Han, Trevor R. Leonardo, Bruna Romana-Souza, Junhe Shi, Shalyn Keiser, Heidi Yuan, Mohamad Altakriti, Matthew J. Ranzer, Sammy Ferri-Borgogno, Samuel C. Mok, Timothy J. Koh, Seok Jong Hong, Lin Chen, Luisa A. DiPietro

**Affiliations:** 1 Center for Wound Healing and Tissue Regeneration, University of Illinois Chicago, Chicago, IL USA; 2 Department of Microbiology and Immunology, College of Medicine, University of Illinois Chicago, Chicago, IL USA; 3 Department of Histology and Embryology, Rio de Janeiro State University, Rio de Janeiro, RJ Brazil; 4 NMPA Key Laboratory for Clinical Research and Evaluation of Traditional Chinese Medicine, Xiyuan Hospital, China Academy of Chinese Medical Sciences, Beijing, China; 5 Department of Surgery, University of Illinois Chicago, Chicago, IL USA; 6 Department of Gynecologic Oncology and Reproductive Medicine, University of Texas MD Anderson Cancer Center, Houston, TX USA; 7 Department of Kinesiology and Nutrition, University of Illinois Chicago, Chicago, IL USA; 8 Department of Surgery, Northwestern University-Feinberg School of Medicine, Chicago, IL USA

**Keywords:** Cell biology, Cell migration, Cell signalling, Cytoskeleton, Mechanisms of disease

## Abstract

Many factors regulate scar formation, which yields a modified extracellular matrix (ECM). Among ECM components, microfibril-associated proteins have been minimally explored in the context of skin wound repair. Microfibril-associated protein 5 (MFAP5), a small 25 kD serine and threonine rich microfibril-associated protein, influences microfibril function and modulates major extracellular signaling pathways. Though known to be associated with fibrosis and angiogenesis in certain pathologies, MFAP5’s role in wound healing is unknown. Using a murine model of skin wound repair, we found that MFAP5 is significantly expressed during the proliferative and remodeling phases of healing. Analysis of existing single-cell RNA-sequencing data from mouse skin wounds identified two fibroblast subpopulations as the main expressors of MFAP5 during wound healing. Furthermore, neutralization of MFAP5 in healing mouse wounds decreased collagen deposition and refined angiogenesis without altering wound closure. In vitro, recombinant MFAP5 significantly enhanced dermal fibroblast migration, collagen contractility, and expression of pro-fibrotic genes. Additionally, TGF-ß1 increased MFAP5 expression and production in dermal fibroblasts. Our findings suggest that MFAP5 regulates fibroblast function and influences scar formation in healing wounds. Our work demonstrates a previously undescribed role for MFAP5 and suggests that microfibril-associated proteins may be significant modulators of wound healing outcomes and scarring.

## Introduction

Wound healing is an evolutionarily conserved process that is essential to the survival of the organism. While some organisms exhibit regenerative capacity following tissue damage or loss, the end result of any significant injury in mammals is nearly always a scar. The regulation of scar formation and fibrosis in skin is multifactorial and involves inflammation and the activation of specific fibroblast subpopulations in the dermis^1,2^. Several studies also link angiogenesis to scar formation, with increased capillary levels linked to worsening scars^3–7^.

The fibroblast is the primary cell type responsible for deposition of new ECM after injury and during the remodeling phases of wound healing, making it a critical component of scar formation^8–10^. Using data from recent studies in our lab involving wound fibroblasts, we have identified MFAP5 as a fibroblast-derived mediator that may significantly impact dermal healing outcomes^11^.

MFAP5, also referred to as Microfibril-associated Glycoprotein 2 (MAGP2), is a microfibril-associated protein that associates with fibrillin to influence microfibril function. Although the composition of the ECM in scar formation has been extensively studied, little prior attention has been paid to the function of microfibril-associated proteins in wound healing. Microfibril-associated proteins are abundant in the ECM of both developing and mature tissues^12^. These proteins play an important role in generating the ECM, thus influencing cellular function.

Among the microfibril-associated proteins, MFAP5 also has the ability to regulate TGFβ bioavailability and Notch signaling via direct binding to Notch receptors or ligands and can bind αvβ3 integrin^12–17^. This suggests that MFAP5 has the potential to alter cellular phenotype by regulating major cellular signaling pathways. There has been recent interest in understanding the role of MFAP5 as it has been found to be highly expressed in stromal fibroblasts of various human cancers, with higher expression linked to enhanced cancer fibrosis, angiogenesis, and chemoresistance^14,15,18–25^. MFAP5 expression is also increased in fibroblasts of fibrotic conditions such as idiopathic pulmonary fibrosis and systemic sclerosis-associated skin and lung fibrosis^26–30^. Taken together, these prior studies suggest that MFAP5 may broadly promote scar formation and fibrosis. To the best of our knowledge, microfibril-associated proteins have not yet been examined in the context of skin repair. The current study examines MFAP5 production and function both in vitro and in vivo. This study demonstrates that MFAP5 may modulate wound angiogenesis and collagen formation, pushing the wound toward a more fibrotic phenotype. In vitro studies show that MFAP5 likely mediates its pro-fibrotic effect on wound healing via modification of fibroblast behavior. These results provide a new understanding of the role of microfibril-associated proteins in skin wounds and skin fibrosis.

## Materials and methods

### Isolation of phagocytic fibroblasts

Previous studies demonstrate that dermal fibroblasts can act as non-professional phagocytes, ingesting apoptotic cells, and that following apoptotic cell engulfment, fibroblasts develop a pro-fibrotic phenotype^11^. Apoptotic human dermal microvascular endothelial cells expressing green fluorescent protein (GFP-HDMECs) were incubated with neonatal human dermal fibroblasts (HDFs) for 8 h as described in our previous publication^11^. HDFs incubated with apoptotic GFP-HDMECs were washed in phosphate-buffered saline (PBS), trypsinized, and centrifuged at 150 × g for 5 min. The cell pellet was resuspended in Dulbecco's Modified Eagle Medium (DMEM) (Mediatech, Manassas, VA) with 20% fetal bovine serum (FBS) (GeminiBio, Sacramento, CA) containing 7-amino-actinomycin D (BD Biosciences, Franklin Lakes, NJ, USA) to exclude dead cells, and then subjected to cell sorting analysis using MoFlo Astrios Cell Sorter (Beckman Coulter, Inc., Indianapolis, IN, USA) to obtain two different subpopulations of HDFs: those that engulfed apoptotic GFP-HDMECs (eApoEC_HDF) and those that did not engulf apoptotic GFP-HDMECs (nApoEC_HDF). Control neonatal human dermal fibroblast cells (CT_HDF) were cultured in parallel with the above experiment without apoptotic endothelial cell treatment. At the time of collection of eApoEC_HDF and nApoEC_HDF, the CT_HDF cells were also washed, trypsinized, and centrifuged in the same manner before cell pellets were snap-frozen using dry ice.

### RNA extraction, library preparation, and RNA-sequencing

Total RNA was isolated and purified with a RNeasy Micro kit (QIAGEN, Hilden, Germany) and treated with DNAse (Promega Corporation, Madison, WI, USA). RNA integrity was checked with Agilent Technologies 2100 Bioanalyzer (Agilent Technologies, Santa Clara, CA). RNA integrity numbers of all samples were between 7.9 and 9.4. A poly (A) RNA-sequencing library was prepared by LC Sciences (Houston, TX, USA) following Illumina’s TruSeq-stranded-mRNA sample preparation protocol (Illumina, San Diego, CA). Poly (A) tail-containing mRNAs were purified using oligo-(dT) magnetic beads with two rounds of purification. After purification, poly (A) RNA was fragmented using divalent cation buffer in elevated temperature. Quality control analysis and quantification of the sequencing library were performed using Agilent Technologies 2100 Bioanalyzer High Sensitivity DNA Chip (Agilent Technologies). Paired-ended sequencing was performed on Illumina’s NovaSeq 6000 sequencing system (Illumina).

### Data processing and differential expression analysis

Cutadapt and perl scripts in house (LC Sciences, Houston, TX, USA) were used to remove any reads containing adapter contamination, low quality bases, and undetermined bases. FastQC was used to verify sequence quality (http://www.bioinformatics.babraham.ac.uk/projects/fastqc/). HISAT2 was used to map reads to the mouse reference genome (ftp://ftp.ensembl.org/pub/release-96/fasta/mus_musculus/dna/). For each sample, mapped reads were assembled using StringTie, and transcriptomes were then merged to reconstruct a comprehensive transcriptome using perl scripts and gffcompare^31^. Raw count data was then input into R, and a DESeqDataSet object was created using the DESeq2 package^32^. Rows with median row counts ≤ 10 were removed and data was annotated with gene symbols and stored for subsequent differential expression analyses. An ANOVA was performed between all groups (eApoEC_HDF, nApoEC_HDF, and CT_HDF) and individual contrasts were also performed: (1) (eApoEC_HDF and nApoEC_HDF) (2) (eApoEC_HDF and CT_HDF) (3) (nApoEC_HDF and CT_HDF). A normalized counts file was generated using the DESeq2 counts() function with normalization = TRUE and saved. For data visualization, count data was log2 transformed using the rlog() function in DESeq2 and saved. To identify secreted or transmembrane proteins whose transcripts were differentially expressed between engulfing and non-engulfing fibroblasts, statistically significant differentially expressed genes (adjusted *p*-value ≤ 0.05) from contrast 1 (eApoEC_HDF and nApoEC_HDF) were intersected with a list of putative secreted or transmembrane proteins generated by Uhlén et al. and plotted via heatmap using heatmap3^33,34^. The adjusted *p*-values (padj) were calculated by a Wald test.

### Animal wound models

All animal procedures and protocols performed were approved by the Institutional Animal Care and Use Committee (IACUC) at the University of Illinois Chicago (UIC). All animal experiments and methods performed were in accordance with the relevant guidelines and regulations. This study was also carried out in compliance with the ARRIVE guidelines. Eight- to 10-week-old female C57BL/6 mice (Jackson Laboratory, Bar Harbor, ME) were housed in groups of five in a temperature-controlled vivarium (22–24 °C) on a 12-h:12-h light–dark cycle and provided with food and water ad libitum. Mice were anesthetized by intraperitoneal injection of ketamine (100 mg/kg) and xylazine (5 mg/kg) solution. The dorsal skin was shaved and cleaned with 70% isopropyl alcohol. Four full-thickness excisional skin wounds were made using a 3- or 5-mm punch-biopsy instrument (Acu Punch, Acuderm Inc, Fort Lauderdale, FL). The excised skin removed during wounding was used as the uninjured base-line normal skin (NS) sample (day 0). On days 1, 3, 5, 7, 14/15, and 21 post-wounding, mice were euthanized by CO_2_ inhalation and cervical dislocation, and the wound tissue was quickly harvested using a standard punch biopsy instrument. Five mice per experimental group were used for each time point post-wounding. A total of 120 mice were used for the excisional wound model assays. For semi-quantitative real-time PCR analysis (RT-PCR), tissues were placed in RNALater® (Sigma-Aldrich) and stored at −20 °C. For immunofluorescent staining, tissues were snap frozen in optimum cutting temperature (OCT) compound (Fisher Scientific, Hampton, NH), cut into 8 µm thick cryosections, and stored at −80 °C. For Masson’s trichrome staining, tissues were stored in 10% neutral buffered formalin solution, paraffin embedded, and sectioned.

A 2 cm full-thickness longitudinal incisional skin wound was made with scissors and closed with two surgical clips because assessment of wound breaking strength may only be performed on incisional skin wound models^35^. The number of mice for each skin wound model was identified by power analysis based on standard deviations from previous studies.

### RT-PCR analysis

For RT-PCR analysis of wound tissue, total RNA from 3- to 5-mm full thickness skin wounds was extracted using TRIzol (Invitrogen, Waltham, MA). One µg total RNA was then treated with DNase (ThermoFisher Scientific, Waltham, MA) and converted to cDNA using a High-Capacity cDNA Reverse Transcription Kit (Invitrogen). Relative *Mfap5* expression was determined by semi-quantitative RT-PCR on a StepOnePlus RealTime PCR System (Applied Biosystems, Waltham, MA) using Power SYBR Green PCR Master Mix (Roche, Basel, Switzerland). The 2^−ΔΔCT^ method was employed to determine the relative expression of target genes^36^. Glyceraldehyde-3-phosphate dehydrogenase (*Gapdh*) was used as a reference gene. NS was used as a baseline for comparisons.

For RT-PCR analysis of cultured fibroblasts, total RNA was extracted using TRIzol (Invitrogen). One µg total RNA was treated with DNase and converted to cDNA as described above. Untreated control fibroblasts were used as a baseline for comparisons. Supplementary Table 1 shows the genes analyzed and primers used for our RT-PCR analyses.

### Treatment of wounds with anti-MFAP5 antibody

Mice were randomly assigned to one of three treatment groups: PBS, unconjugated mouse IgG isotype control antibody (ThermoFisher Scientific, Catalog # 31,903), or an anti-MFAP5 monoclonal antibody. Anti-MFAP5 monoclonal antibody (clone 130A), cross-reactive to both human and mouse MFAP5, was generated in mice as previously described^20^. Initially, each mouse excisional skin wound was treated topically with 40 µl of PBS, 1.5 µg of mouse IgG control (ThermoFisher Scientific), or 1.5 µg of anti-MFAP5 monoclonal antibody suspended in 40 µL PBS. Subsequent treatments were performed via subcutaneous injection of 40 µL PBS or 1.5 µg of either anti-MFAP5 monoclonal or control antibody suspended in 40 µL PBS under each of the four wounds on days 3, 6, 9, 12, and 15 post-wounding. Wounds were photographed every other day during healing, and the size of each wound was measured using AxioVision software (Zeiss, Oberkochen, Germany). Four wounds from each mouse were averaged to produce one unique value per animal. The percentage of open wound area, as compared to the original wound area, was calculated at each time point. Wound tissue from each mouse was collected on days 7, 14, and 21 post-wounding as described above for our excisional wound model assays. Ten mice per experimental group were used for each time point post-wounding for a total of 90 mice used for the in vivo MFAP5 antibody neutralization assays.

For the incisional skin wounds, wounds were treated topically with 160 µL PBS, 6 µg mouse IgG control (ThermoFisher Scientific), or 6 µg anti-MFAP5 monoclonal antibody suspended in 160 µL PBS at the time of wound placement. Wounds subsequently received subcutaneous injection of 160 µL PBS or 6.0 µg of either anti-MFAP5 monoclonal or control antibody suspended in 160 µL PBS across the whole wound on days 3, 6, 9, 12, and 15 post-wounding. On days 14 and 21 post-wounding, mice were euthanized, and the wound tissue was collected for assessment of wound breaking strength. Five mice per experimental group were used for each time point. A total of 30 mice were used for the wound breaking strength assay.

### Masson’s trichrome stain

Masson’s trichrome stain was performed as described in our previous publications^37,38^. Images were obtained at 20X magnification. To quantify collagen deposition, the percent of blue-stained collagen area within the wound bed was measured by ImageJ software^38^ and normalized to NS.

### Indirect immunofluorescent staining

Cryosections of mouse skin wound samples (8 μm thick) were air-dried, rehydrated in PBS, and fixed in cold acetone. Sections were washed with PBS and blocked with 10% goat serum (Sigma-Aldrich) at room temperature prior to incubation with primary antibodies. Cryosections were incubated with rabbit anti-mouse MFAP5 antibody (MyBioSource, San Diego, CA, Catalog # MBS7004362) at 1:200 dilution or rabbit IgG control (Jackson ImmunoResearch Laboratories, West Grove, PA) at the same concentration overnight at 4 °C and then with Alexa Fluor 594 goat anti-rabbit IgG (Invitrogen) at 1:1000 dilution. To detect blood vessel content in healing mouse skin wounds treated with PBS, mouse IgG control (ThermoFisher Scientific), or anti-MFAP5 monoclonal antibody, cryosections were incubated with rat anti-mouse CD31 (BD Pharmingen, San Diego, CA, Catalog # 557,355) at 1:1600 dilution. Sections were then washed with PBS and incubated with secondary antibodies: Alexa Fluor 488 goat anti-rat IgG (Invitrogen) or Alexa Fluor 594 goat anti-rat IgG (Invitrogen) at 1:1000 dilution. After washing with PBS, samples were mounted with 50% glycerol in PBS containing DAPI (Sigma-Aldrich) for nuclear counterstaining. All primary and secondary antibody incubations occurred for 1 h at room temperature unless otherwise stated. All images were taken under 20X magnification using the Axioskop 40 fluorescence microscope (Zeiss). To quantify vascularity, the percent of CD31 + areas was quantified by ImageJ software^39^ and normalized to the percent of CD31 + areas in NS.

To detect MFAP5 in human keloid tissue and NS, 5 μm thick paraffin-embedded sections were used. Sections were de-paraffinized and rehydrated using a standard protocol. Antigen retrieval was performed using Epitope Retrieval Solution (IHC world, Ellicott City, MD) and a steamer according to manufacturer’s protocol. Following antigen retrieval, sections were blocked with 10% normal goat serum (Sigma Aldrich), incubated with rabbit anti-human MFAP5 polyclonal antibody (Thermo-Fisher Scientific, Catalog # PA5-14,204) at 1:100 dilution or rabbit IgG control (Jackson ImmunoResearch Laboratories) at the same concentration, washed with PBS, and then incubated with Alexa Fluor 594 goat anti-rabbit IgG (Invitrogen) before mounting. Images were taken as described above.

### Assessment of wound breaking strength

Two wound-pelts isolated from the incisional wound per mouse were subjected to wound breaking strength analysis using a motorized tensiometer (Mark-10, Copiague, NY) as described previously^35,40^. The average was recorded as the wound breaking strength for that individual animal. NS tissue was also tested. Relative wound breaking strength is expressed as a percentage of NS strength.

### Gene ontology analysis of fibroblast subpopulations

Publicly available supplementary datasets from a previous study were reanalyzed^2^. Specifically, Supplementary Data Files 1 and 2 from Guerrero-Juarez et al., were used for our study^2^. In this prior study, single-cell RNA-sequencing of unsorted cells from mouse skin wounds at 12 days post-wounding (GSE113605) was performed^2^. Wound cell types were identified by their distinct gene signatures and wound fibroblasts specifically underwent principal component analysis (PCA) and differential expression analysis via the Seurat package in R^2^. Wound fibroblasts were then subsequently divided into 12 distinct sub-clusters designated as sC1 through sC12 based on these results^2^. All genes listed for each sub-cluster had a positive log2foldchange > 0.25. GO enrichment analysis was performed using the R package EnrichR with the differentially expressed genes listed for individual wound fibroblast sub-clusters. Genes were annotated to biological processes (BP) and cellular components (CC). GO terms with a padj < 0.05 following Kolmogorov–Smirnov testing were considered statistically significantly enriched terms.

### Analysis of fibroblast migration, proliferation, gel contraction, and gene expression

Human neonatal skin fibroblasts immortalized with hTERT (BJ-5ta) (ATCC, Manassas, VA) were cultured in a 4:1 mixture of DMEM (Mediatech) and Medium 199 (Sigma-Aldrich) supplemented with 0.01 mg/ml hygromycin B (Sigma-Aldrich) and 10% FBS (GeminiBio). For in vitro experiments, growth media containing 10% FBS (GeminiBio) was considered the normal serum condition. Cells were used once they reached 80%-90% confluence and were incubated at 37 °C in an atmosphere of 5% CO_2_. All experiments were repeated at least 3–6 times.

To assess the effects of exogenous MFAP5 on fibroblast migration, fibroblasts were grown in a 3-well culture-insert (Ibidi USA Inc, Fitchburg, WI) and treated with mitomycin-C (5 μg/ml) (Sigma-Aldrich) for 1 h. Afterwards, the insert was removed, leaving a 500 µm wide open space. Cells were washed with PBS and treated with fresh media supplemented with varying concentrations of recombinant MFAP5 (rMFAP5) (R&D Systems, Minneapolis, MN) dissolved in PBS: 5, 20, 100, or 200 ng/mL rMFAP5 (R&D Systems). Media without rMFAP5 (R&D Systems) was used as a control. The opened area was photographed and measured using ImageJ software (US NIH, Bethesda, MD)^39^. Rate of migration is expressed as a percentage of the original uncovered area for each time point.

To evaluate cell proliferation, 3,000 fibroblasts were seeded in each well of a 96-well plate and treated with 200 or 400 ng/mL rMFAP5 (R&D Systems). Proliferation was assessed 12, 24, and 36 h later using an MTS Cell Proliferation Assay Kit (Abcam, Cambridge, MA).

To measure collagen gel contraction, fibroblasts were pre-treated with 200 ng/mL rMFAP5 (R&D Systems) for 24 h. Subsequently, cells were trypsinized and embedded in a collagen type I solution (BD Biosciences) containing fresh media supplemented with 200 ng/mL rMFAP5 (R&D Systems) and transferred to a 24-well plate^41^. Non-pretreated fibroblasts and collagen solutions without any rMFAP5 supplementation were used as a control. The gel was then photographed at 0, 24, 48, and 72 h, and gel area was measured using ImageJ software^39^. Collagen contractility is expressed as the percent area of original collagen gel surface area for each time point.

To assess the expression of genes linked to fibrosis^42^ in response to rMFAP5 treatment, fibroblasts were grown in a 12-well plate, and fresh media supplemented with 200 ng/mL rMFAP5 (R&D Systems) was added. Twenty-four h later, RT-PCR for select genes (Supplementary Table 1) was performed as described above. For all experiments, media without rMFAP5 was used as the control.

To account for potential serum MFAP5 affecting fibroblast response to rMFAP5 treatment, all in vitro assays were repeated under low serum conditions^15,20^. Fibroblasts were cultured in normal serum until 80–90% confluency, then switched to a low serum, 1% FBS (GeminiBio), growth media and incubated overnight. The following day, fibroblasts underwent the same in vitro assays described above for normal serum conditions. For the gene expression assay under low serum, cells were treated with rMFAP5 (R&D Systems) for only 6 h before total RNA was extracted. Overall, the in vitro assays performed in low serum had similar results to the in vitro assays performed in normal serum.

### Analysis of fibroblast MFAP5 expression following TGF-ß1 treatment

For all experiments, cells were grown to 70–80% confluence in normal growth media and then switched to low serum media. BJ-5ta (ATCC) and primary adult fibroblasts (PromoCell, Heidelberg, Germany) were grown in a 6-well plate and treated with 10 ng/mL TGF-ß1 (PeproTech, Cranbury, NJ). Thirty-six hours after TGF-ß1 treatment, RT-PCR analysis for *MFAP5* expression was performed as described above.

For immunohistochemical assessment of MFAP5 protein expression in response to TGF-ß1, BJ-5ta and primary adult fibroblasts were cultured in an EZ slide 4 well chamber slide (Fisher Scientific) and then treated with 10 ng/mL TGF-ß1. After 36 h, cells were washed with PBS, fixed with 4% paraformaldehyde, permeabilized with 0.5% triton, and blocked with 10% normal goat serum (Sigma-Aldrich). Subsequently, cells were incubated with rabbit anti-MFAP5 antibodies (Abcam, Catalog # ab203828) at 1:250 dilution overnight at 4 °C and then Alexa Fluor 594 goat anti-rabbit IgG (Invitrogen) at 1:1000 dilution for 1 h at room temperature. Samples were mounted with 50% glycerol in PBS containing DAPI (Sigma-Aldrich) for nuclear counterstaining. The images were acquired at 20× magnification using a fluorescence microscope (Axioskop 40) and AxioCam MRc camera (Zeiss). Images were then processed with Leica Processing software. Total intensity and pixel area of red fluorescence was measured with ImageJ software (NIH)^39^. Total intensity was normalized to pixel area to generate relative MFAP5 fluorescence.

### Keloid sample collection

Keloid samples were collected from the discarded tissue of three male patients undergoing keloid scar revision surgery on the ear. NS was collected from a male patient also undergoing keloid scar revision surgery on the ear. Informed consent was obtained from each patient. Tissue collection was approved by the Institutional Review Board of University of Illinois Chicago and of Northwestern University. All methods and experiments performed using these human samples were in accordance with the relevant IRB guidelines and regulations. Collected tissues were fixed in 10% neutral buffered formalin solution, paraffin embedded, and sectioned using a standard protocol.

### Gene and protein nomenclature

Mouse gene and protein nomenclature follows the guidelines outlined by the Mouse Genome Database and the International Committee on Standardized Genetic Nomenclature for Mice^43^, while human gene and protein nomenclature follows the guidelines outlined by the HUGO Gene Nomenclature Committee^44^.

### Statistical analysis

Data were expressed as mean ± standard deviation (SD), and their normality was evaluated using the Kolmogorov–Smirnov or Shapiro–Wilk tests. Statistical comparisons were performed using a two-tailed unpaired t-test with Welch’s correction, or a one- or two-way ANOVA followed by two-stage linear step-up procedure of Benjamini, Krieger, and Yekutieli post-hoc testing using GraphPad Prism version 8.0 (GraphPad, San Diego, CA). *P*-values less than 0.05 were considered statistically significant.

## Results

### MFAP5 expression is upregulated in phagocytic fibroblasts

**Figure 1:**
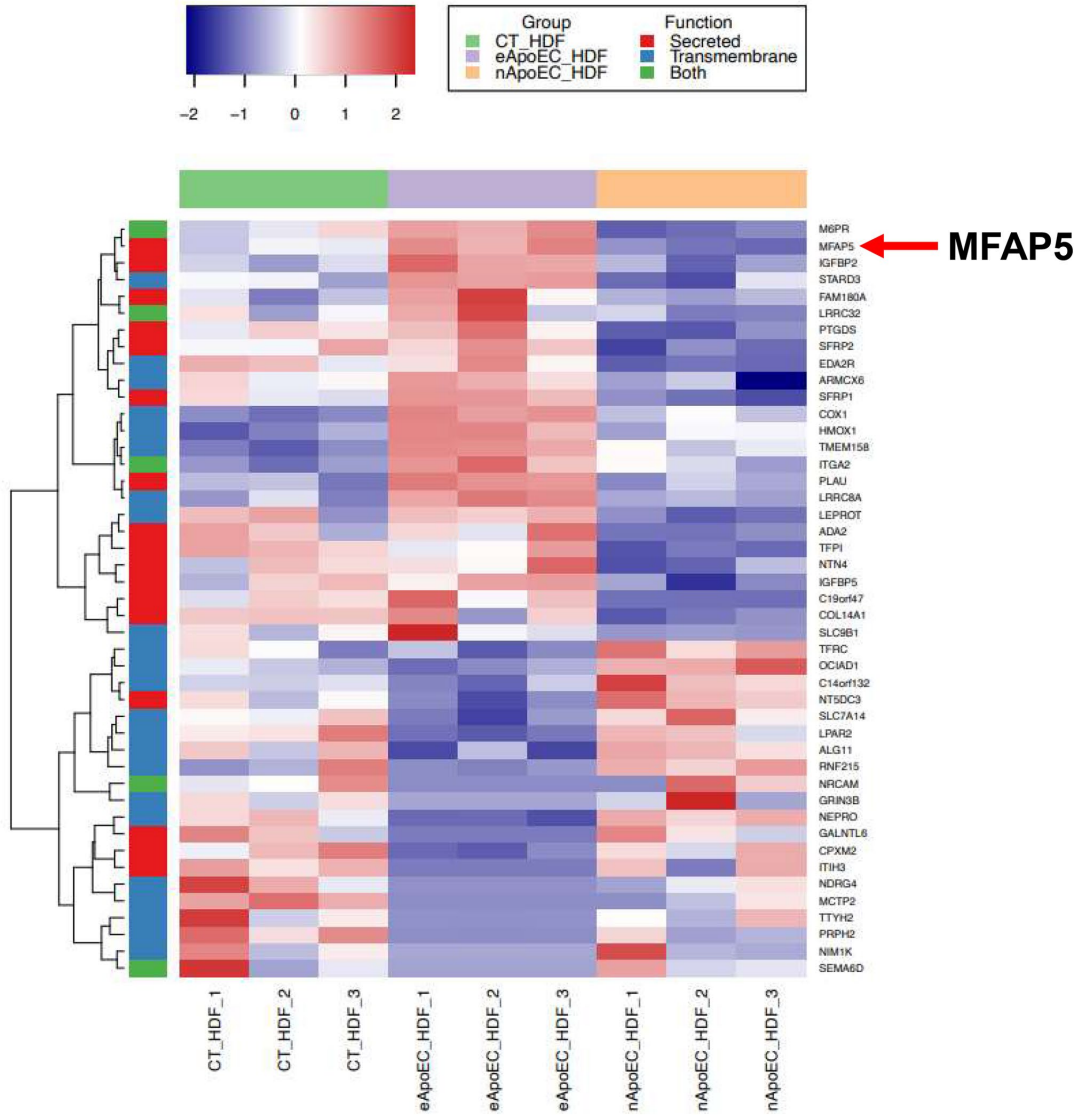
MFAP5 is upregulated in phagocytic fibroblasts. Heatmap of differentially expressed genes between phagocytic and non-phagocytic fibroblasts incubated with GFP-HDMECs (padj ≤ 0.05) that were identified as putative secreted proteins (red), transmembrane proteins (blue) or both (green). Genes were hierarchically clustered using the complete agglomeration method. Each row represents a gene, and each column represents an individual sample in the following groups: control fibroblasts (CT_HDF), fibroblasts phagocytosing apoptotic endothelial cells (eApoEC_HDF), and non-phagocytosing fibroblasts exposed to apoptotic endothelial cells (nApoEC_HDF). N = 3 for each group. The data used was log2 transformed count data. Adjusted *p*-values were calculated with a Wald test.

Recent studies have shown that wound fibroblasts can phagocytize apoptotic cells, and that these phagocytic fibroblasts adopt a pro-fibrotic phenotype^11^. To examine the unique proteins that are produced by phagocytic fibroblasts, an RNA-sequencing approach was employed. Apoptotic endothelial cells were added to fibroblast cultures for 8 h, and FACS was performed to isolate phagocytic and non-phagocytic fibroblast populations^11^. These two groups of fibroblasts as well as normal fibroblasts with no added endothelial cells (CT_HDF) were subjected to RNA-sequencing and differential expression analyses. The PCA plot is shown in Supplementary Fig. 1. To identify secreted or transmembrane proteins, statistically significant (padj ≤ 0.05) differentially expressed transcripts between the engulfing and non-engulfing fibroblasts were intersected with a list of putative secreted and transmembrane proteins generated by Uhlén, M. et al., (2015) and plotted via heatmap (Fig. 1)^33^. When compared to the CT_HDF and non-phagocytic fibroblast group (nApoEC_HDF), MFAP5 was found to be one of the top differentially expressed genes in the phagocytic fibroblast group (eApoEC_HDF) that was also a putative transmembrane and secreted protein. Since previous work has implicated MFAP5 in fibrotic pathologies, it was further examined as a potential pro-fibrotic mediator.

### MFAP5 is upregulated in the proliferative and remodeling phases of skin wound healing

**Figure 2:**
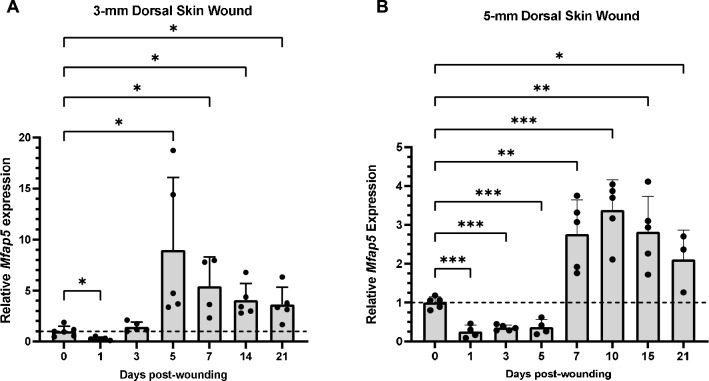
*Mfap5* expression is upregulated during the proliferative and remodeling phases of wound healing*.* RT-PCR of full thickness 3-mm (**A**) and 5-mm (**B**) dorsal mouse wound tissue over the time course of healing. Mice used for this experiment were all female. *Mfap5* expression was normalized to *Gapdh* expression and is expressed as 2^−ΔΔCT^. Bars indicate mean ± SD. N = 3–6 with each dot representing a biological replicate that consists of 3 technical replicates at each time point. Day 0 post-wounding samples are normal skin. One-way ANOVA with two-stage linear step-up procedure of Benjamini, Krieger, and Yekutieli post-hoc testing (vs 0), * = *p* < 0.05, ** = *p* < 0.01, *** = *p* < 0.001.

Since *MFAP5* expression was found to be upregulated in pro-fibrotic phagocytic fibroblasts, we next examined whether MFAP5 is modulated during skin wound healing. *Mfap5* expression was examined in 3- and 5-mm skin wounds of C57BL/6 J mice. In 3-mm skin wounds, *Mfap5* expression is significantly increased 5 days post-wounding and remained significantly elevated through day 21 (Fig. 2A). In 5-mm skin wounds, *Mfap5* expression increased 7 days post-wounding and also remained significantly elevated through day 21 (Fig. 2B). Interestingly, *Mfap5* expression was significantly decreased at 1 day post-wounding in the 3-mm skin wound and between 1 and 5 days post-wounding in the 5-mm skin wound (Fig. 2). These results demonstrate that *Mfap5* expression is markedly increased at sites of skin injury, with peak expression occurring during the later proliferative and remodeling phases of wound healing.

**Figure 3:**
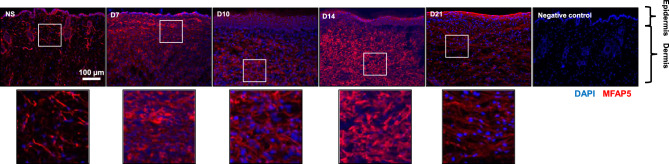
MFAP5 is predominantly expressed extracellularly in unwounded normal skin and in dermal wounds. Representative photomicrographs of fluorescence microscopy images of MFAP5 (red) immunohistochemical staining of normal skin (NS) and 3-mm full thickness dorsal mouse wounds from female mice at 7, 10, 14, and 21 days post-wounding. Nuclei in blue (DAPI). Magnified portions of each original photomicrograph are depicted by the white box and shown below the original.

To determine the localization of MFAP5 in wounds, wound sections of C57BL/6 J mice were examined using indirect immunofluorescence. Unwounded NS predominantly exhibited extracellular MFAP5 staining in the dermis (Fig. 3). Throughout healing, dermal wound beds also primarily exhibited positive extracellular MFAP5 staining (Fig. 3). In addition to the dermal staining, MFAP5 was unexpectedly prevalent in the epidermis (Fig. 3).

To examine MFAP5 localization and expression in human scars, indirect immunofluorescence was performed on human keloids and NS. MFAP5 was strongly expressed in keloids and was localized to both the dermis and epidermis (Supplementary Fig. 2). NS also had strong MFAP5 expression, but mainly in the epidermis (Supplementary Fig. 2). Taken together, these results demonstrate that MFAP5 is expressed in keloid scars.

### Neutralizing MFAP5 inhibits collagen deposition and angiogenesis in skin wounds

**Figure 4:**
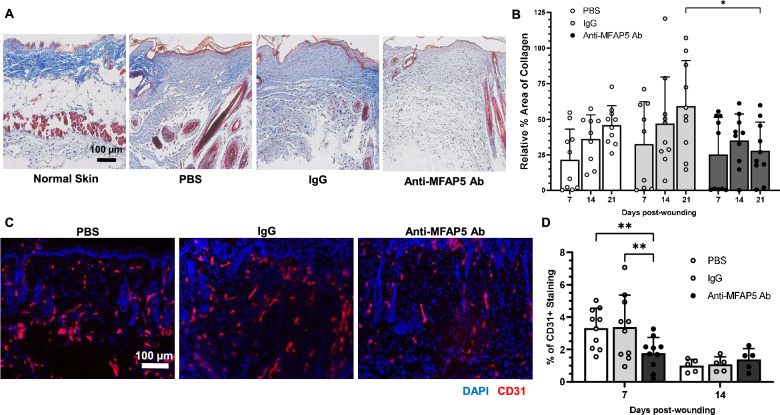
Mouse wounds treated with anti-MFAP5 antibodies exhibit decreased collagen deposition and angiogenesis. Collagen content and angiogenesis in full thickness 5-mm dorsal mouse wounds treated with PBS, IgG, or anti-MFAP5 antibody was assessed by Masson's trichrome and immunofluorescence staining, respectively. (**A**) Representative photomicrographs of 5-mm full thickness dorsal mouse wounds at 21 days post-wounding. (**B**) Quantitative collagen deposition in mouse wounds expressed as percent area-stained blue at days 7, 14, and 21 post-wounding, relative to normal skin (NS). N = 9–10 in each group. (**C**) Representative photomicrographs of fluorescence microscopy images of CD31 (red) staining in tissue from 7 days post-wounding. Nuclei in blue (DAPI). (**D**) Vessel density in tissue at days 7 and 14 post-wounding following treatments expressed as the percent CD31 positive area in wounds relative to NS. N = 5–10 in each group. Bars on all graphs indicate mean ± SD. * = *p* < 0.05, ** = *p* < 0.01. Two-way ANOVA with two-stage linear step-up procedure of Benjamini, Krieger, and Yekutieli post-hoc testing (vs. PBS or IgG).

To evaluate whether MFAP5 is involved in collagen deposition in skin wound healing, MFAP5 was neutralized by treatment of murine wounds with an anti-MFAP5 antibody. No differences in rate of wound closure were observed in mouse wounds treated with anti-MFAP5 antibodies as compared to control wounds (Supplementary Fig. 3). There were also no statistically significant differences observed in the wound breaking strength of mouse wounds treated with anti-MFAP5 antibodies as compared to control wounds (Supplementary Fig. 4). However, as compared to control wounds treated with mouse IgG control antibodies, wounds treated with anti-MFAP5 antibodies exhibited a significant decrease in collagen deposition at day 21 post-wounding (Fig. 4A,B). When wound angiogenesis was quantified, wounds treated with anti-MFAP5 antibodies showed significantly less angiogenesis at day 7 post-wounding as compared to controls (Fig. 4C,D), a difference that resolved by day 14 post-wounding (Fig. 4D). These results suggest MFAP5 promotes collagen deposition and angiogenesis during wound healing in vivo.

### MFAP5 is upregulated in fibroblast subpopulations in wounds

**Figure 5:**
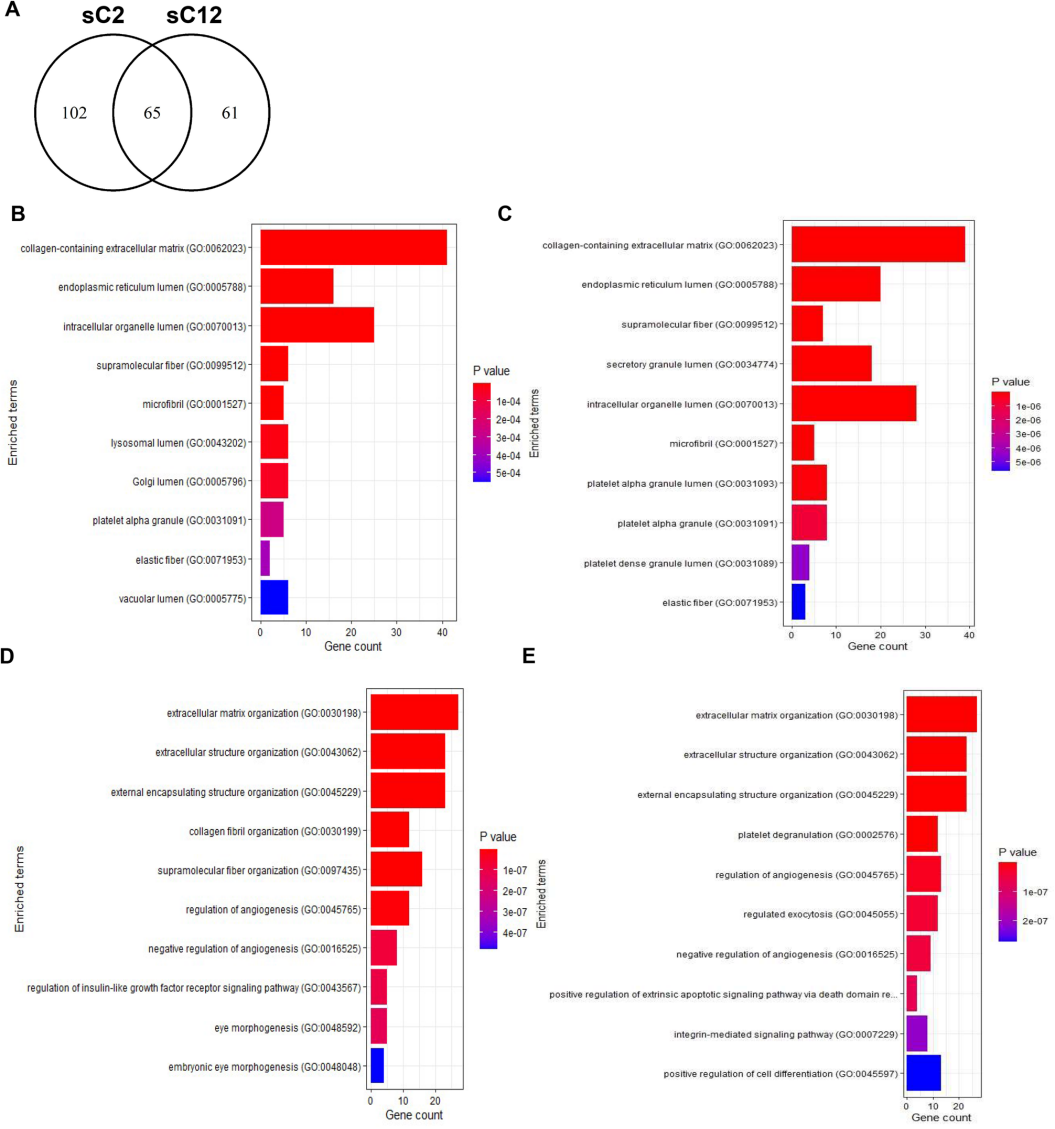
Top 10 cellular components (CC) and biological processes (BP) gene ontology (GO) terms of wound fibroblasts that differentially express *Mfap5 *in vivo identify functions likely related to ECM synthesis and organization. (**A**) Venn diagram comparing the number of distinct and shared differentially expressed genes for sC2 and sC12. Top 10 significantly enriched (padj < 0.05) GO terms for CC of wound fibroblast subpopulation sC2 (**B**) and sC12 (**C**). Top 10 significantly enriched (padj < 0.05) GO terms for BP of wound fibroblast subpopulation sC2 (**D**) and sC12 (**E**). Adjusted *p*-values for GO terms were determined by Kolmogorov–Smirnov testing.

To examine if certain populations of wound fibroblasts might uniquely express MFAP5, we took advantage of available supplementary datasets generated by Guerrero-Juarez et al.^2^. This previous study included an analysis of single-cell RNA-sequencing data of unsorted cells from mouse skin wounds at 12 days post-wounding. To determine if fibroblasts were a major source of MFAP5, we analyzed Supplementary Data File 1 from the Guerrero-Juarez et al. study, which lists the differentially expressed gene signatures for each captured wound cell. To determine if specific fibroblast subpopulations differentially expressed *Mfap5* during wound healing in vivo, we analyzed Supplementary Data File 2, which lists the differentially expressed gene signatures for each wound fibroblast sub-cluster. Analysis of these data sets showed that fibroblasts were the main expressors of *Mfap5* and that *Mfap5* was significantly expressed in specific sub-clusters of wound fibroblasts. Among the 12 subpopulations, sC2 and sC12 differentially expressed *Mfap5*. SC2 had 167 differentially expressed gene signatures, and sC12 had 126 differentially expressed gene signatures (Fig. 5A). Between sC2 and sC12, there were 65 shared differentially expressed gene signatures (Fig. 5A). To gain insight into the functions of the wound fibroblast subpopulations that differentially express *Mfap5*, gene ontology (GO) enrichment analysis was performed on those subpopulations’ list of differentially expressed gene signatures via the EnrichR package in R version 4.2.1^45–47^. GO term enrichment analysis showed a number of significant GO terms (padj < 0.05) enriched for BP and CC in wound fibroblast sub-clusters, sC2 and sC12. The top 10 GO terms for CC and BP, ranked by padj, are shown in Fig. 5B–E. ECM component related CC terms such as collagen-containing ECM, supramolecular fiber, microfibril, and elastic fiber were significantly upregulated in sC2 and sC12 (Fig. 5B,C). ECM organization related BP terms such as ECM organization and extracellular structure organization were significantly upregulated in sC2 and sC12 (Fig. 5D,E). Genes annotated to each of the top 10 CC and BP GO terms for sC2 and sC12 are listed in Supplementary Tables 2–5. Taken together, our analysis shows that fibroblasts are a major source of MFAP5 in healing wounds and that the two subpopulations that differentially express *Mfap5* are likely to be important contributors to the synthesis and organization of ECM during wound healing.

### MFAP5 regulates fibroblast phenotype and gene expression profile in vitro

Given that upregulation of *MFAP5* is associated with a pro-fibrotic phenotype in phagocytic fibroblasts and that MFAP5 is upregulated in mouse wounds and localizes to dermal scar ECM, we wanted to evaluate whether in vitro treatment with exogenous rMFAP5 could stimulate a pro-fibrotic phenotype in fibroblasts. To investigate the effect of MFAP5 on fibroblast function, normal human dermal fibroblasts were treated with rMFAP5, and characteristics known to be linked to a pro-fibrotic phenotype (cell migration, cell proliferation, contractile activity, ECM related gene expression) were evaluated. Because 200 ng/mL rMFAP5 and 24 h incubation elicited the most significant response in fibroblasts for migration, those conditions were used as the baseline concentration for all in vitro assays.

**Figure 6:**
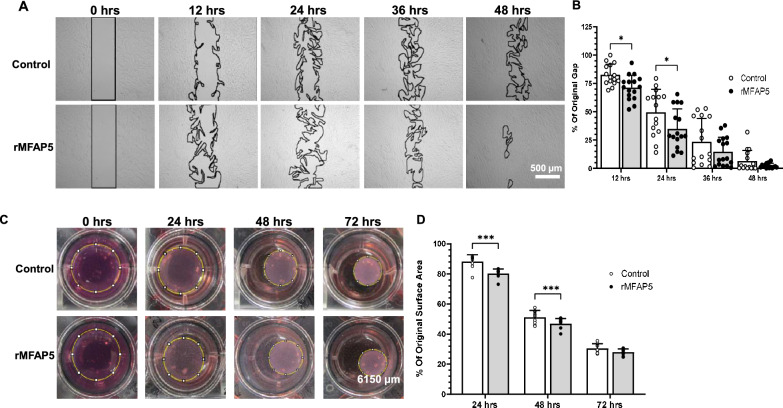
Exogenous MFAP5 treatment increases fibroblast migration and collagen gel contraction in vitro. (**A**) Representative photos of the fibroblast cell migration assay in media supplemented with or without 200 ng/mL recombinant MFAP5 (rMFAP5). Areas not covered by cells are outlined by a black line. (**B**) Rate of cell migration, expressed as a percentage of the original uncovered area. N = 15–16. (**C**) Representative photos of the collagen gel contraction assay for dermal fibroblasts cultured in media with or without 200 ng/mL rMFAP5. Gel area is depicted by a yellow line. Scale bar = 6150 μm. (**D**) Rate of gel contraction, expressed as a percentage of the original gel surface area. N = 9. * = *p* < 0.05, ** = *p* < 0.01, *** = *p* < 0.0001. Two-way ANOVA with two-stage linear step-up procedure of Benjamini, Krieger, and Yekutieli post-hoc testing (vs. Control).

Treatment of fibroblasts with rMFAP5 caused significantly increased rates of cell migration at 12 and 24 h post-treatment under both normal and low serum conditions (Fig. 6A,B, and Supplementary Fig. 5). However, treatment of fibroblasts with rMFAP5 did not significantly change cell proliferation in either normal serum (Supplementary Fig. 6A) or low serum conditions (Supplementary Fig. 6B).

To examine the effect of rMFAP5 on contractile activity, fibroblasts were pre-treated with rMFAP5, embedded in a collagen type I gel supplemented with rMFAP5, and collagen gel contraction was measured over time. Treatment with rMFAP5 led to a significant increase in the rate of collagen gel contraction at 24 and 48 h (Fig. 6C,D). The effects of rMFAP5 on contractile activity were similar under both normal (Fig. 6C,D) and low serum conditions (Supplementary Fig. 7).

**Figure 7:**
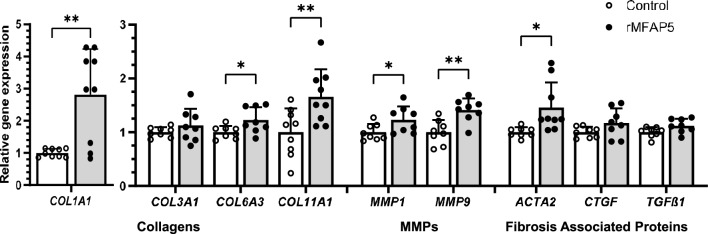
Relative gene expression levels of pro-fibrotic genes and ECM proteins in dermal fibroblasts treated with exogenous MFAP5 in vitro. RT-PCR performed on fibroblasts cultured with or without 200 ng/mL recombinant MFAP5 (rMFAP5) for 24 h. Gene expression levels were normalized to *GAPDH* and expressed as 2^−ΔΔCT^. Bars indicate mean ± SD. N = 8–9, with each dot representing a biological replicate that consists of 3 technical replicates. * = *p* < 0.05, ** = *p* < 0.01. Two-tailed unpaired t-test with Welch’s correction.

To investigate if rMFAP5 influences collagen synthesis, the relative gene expression of *COL1A1, COL3A1, COL6A3,* and *COL11A1* was measured by RT-PCR. Gene expression of *COL1A1, COL6A3,* and *COL11A1* was significantly enhanced in fibroblasts treated with rMFAP5 for 24 h when compared to untreated control cells (Fig. 7). To determine if rMFAP5 also promoted ECM remodeling, relative *MMP1* and *MMP9* gene expression levels were measured. Gene expression of *MMP1* and *MMP9* was significantly increased in fibroblasts cultured with rMFAP5 for 24 h when compared to untreated control (Fig. 7). The effect of rMFAP5 on the expression of genes associated with fibrosis, including *ACTA2, CTGF,* and *TGFß1*, was also assessed. Treatment of fibroblasts with rMFAP5 for 24 h exhibited significantly increased *ACTA2* expression (Fig. 7). Similar findings were seen under low serum conditions, but with changes in gene expression occurring much earlier at 6 h post-rMFAP5 treatment (Supplementary Fig. 8). Interestingly, under low serum conditions, rMFAP5 treatment decreased relative *MMP9* expression in fibroblasts (Supplementary Fig. 8).

**Figure 8:**
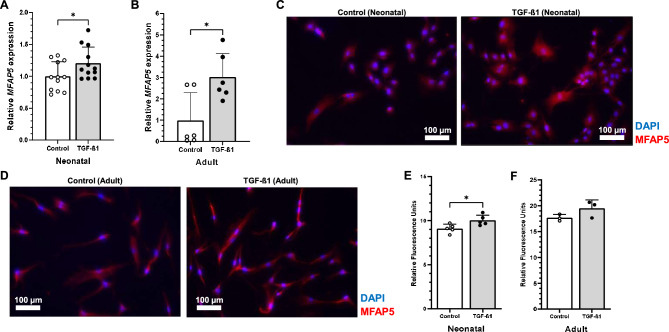
TGF-ß1 increases relative MFAP5 gene and protein expression in neonatal and adult dermal fibroblasts in vitro. RT-PCR performed on neonatal (**A**) and adult (**B**) fibroblasts treated with or without 10 ng/mL TGF-ß1 for 36 h. *MFAP5* expression was normalized to *GAPDH* expression and is expressed as 2^−ΔΔCT^. N = 6–9, with each dot representing a biological replicate that consists of 3 technical replicates. Representative images of fluorescence microscopy of MFAP5 (red) in TGFß-1 treated or control neonatal (**C**) and adult (**D**) fibroblasts. Nuclei in blue (DAPI). Relative fluorescence of MFAP5 in TGF-ß1 treated or control neonatal (**E**) and adult (**F**) fibroblasts. N = 3–5. Bars on all graphs indicate mean ± SD. * = *p* < 0.05. Two-tailed unpaired t-test with Welch’s correction was used for (**A**, **B**, **E**, and **F**).

To examine whether a fibrotic environment might modulate MFAP5 production, fibroblasts were treated with TGF-β1. Neonatal and adult fibroblasts treated with TGF-β1 significantly increased *MFAP5* expression at 36 h (Fig. 8A,B). Additionally, immunocytochemical analysis showed significantly increased MFAP5 protein expression in neonatal fibroblasts treated with TGF-ß1 as compared to control (Fig. 8C–F).

Taken together, these data suggest that exogenous rMFAP5 promotes a pro-fibrotic phenotype in dermal fibroblasts in vitro and that TGF-β1 can increase MFAP5 gene and protein expression.

## Discussion

A number of studies have examined the mechanisms that cause scarring and fibrosis following skin injury in mammals. While the ECM composition in normal and scar dermis has been well studied for collagen content and architecture, studies of microfibrils and their components in healing skin wounds are rare^48,49^. A role for the microfibril-associated glycoprotein MFAP5 in wound healing and wound fibrosis was first suggested when we identified MFAP5 as a molecule that is significantly upregulated in phagocytic fibroblasts. Phagocytic fibroblasts are a unique population in wounds and have been suggested to support scar formation versus regenerative outcomes^11^.

As a microfibrillar protein, the examination of MFAP5 in wound healing is relatively unique, as this class of protein has not been explored in the context of skin wound repair. Of relevance to the current study, research in other systems suggests that MFAP5 has additional roles and interactions beyond those proposed for microfibrils. MFAP5 may bind growth factors like TGF-ß, Notch receptors and ligands, and αvß_3_ integrin^12,14,17,18^. More recently, MFAP5 has been shown to be expressed at high levels in stromal fibroblasts of various human cancers, with higher expression linked to poorer outcomes^14,18–22,24,25^. In these cancers, MFAP5 alters cellular phenotype in various cell types in the tumor microenvironment to promote fibrosis, angiogenesis, and chemoresistance. Though MFAPs have now been relatively well studied in various cancer types and human diseases, little is known regarding their role in wound healing^50–52^.

Our work fills this gap in knowledge, showing that MFAP5 is upregulated during the later phases of wound healing. MFAP5 is likely most active during the proliferative and tissue remodeling phases, when angiogenesis and scar formation occur^4,8^. Indirect immunofluorescent staining demonstrated that MFAP5 was present in the dermis and epidermis in unwounded NS and wound tissue throughout healing. MFAP5 was also expressed in keloids with primarily dermal and epidermal staining. Our staining results in wound, keloid, and normal skin tissue coincide with previous work studying expression patterns of MFAP5 in various cancers and the predicted locations by Human Protein Atlas (proteinatlas.org), which also suggests that fibroblasts are a major source of MFAP5^15,19,22,33,53–56^. Future studies involving co-staining of MFAP5 with a cytoplasmic marker, such as vimentin, which also functions as a fibroblast marker, could be performed to determine if MFAP5 is also found intracellularly in fibroblasts of unwounded NS and wound tissue.

Surprisingly, our studies also demonstrated prominent epidermal staining of MFAP5. This finding suggests that keratinocytes may be a producer of MFAP5, a discovery that will require further investigation and validation. Taken together, though, pathologic overexpression of MFAP5 in both fibroblasts and keratinocytes may contribute to keloid formation. This finding is in part supported by recently published single-cell RNA-sequencing and spatial transcriptomics studies which show that keloid fibroblasts significantly overexpress MFAP5 as compared to fibroblasts from NS^57,58^.

Our analysis of prior data from mouse skin wounds revealed that particular sub-clusters of wound fibroblasts are likely the major source of MFAP5. Our finding that mouse wounds treated with anti-MFAP5 antibodies exhibited significantly decreased angiogenesis and collagen content agrees with previous findings demonstrating that antibody neutralization of MFAP5 reduced fibrosis in mouse tumors^20^. Together, all of these studies point toward a role for MFAP5 in wound healing and skin scar formation.

The in vitro studies here suggest several mechanisms by which MFAP5 might affect wound healing outcomes. Human dermal fibroblasts treated with rMFAP5 demonstrated increased gene expression of *COL1A1* and *COL11A1,* as well as increased *COL6A3*, *MMP1,* and *MMP9* expression. These results are similar to findings in cancer associated fibroblasts, which demonstrated an increase in *COL1A1* and *COL11A1* expression following rMFAP5 treatment^20^.

In considering the cellular response to MFAP5, MFAP5 is capable of binding to fibroblast αvß_3_ integrin through its RGD motif^12^. Activation of αvß_3_ integrin has been shown to subsequently stimulate collagen gene expression as well as to drive fibroblast contraction and ECM stiffening in progressive fibrosis^12,18,59,60^. Perhaps during wound healing, MFAP5 binds fibroblast αvß_3_ integrin to promote ECM deposition and remodeling. This correlates with our observation that MFAP5 expression significantly increases during the later phases of wound healing, where fibroblasts produce a fibrous scar and differentiate into myofibroblasts to exert contractile force. In congruence with this idea, dermal fibroblasts treated with rMFAP5 exhibited significantly enhanced expression of *ACTA2*, a marker of myofibroblast differentiation, and promoted fibroblast collagen gel contraction. Further studies involving knockdown of fibroblast αvß_3_ integrin prior to rMFAP5 treatment will be necessary to determine if MFAP5 induces fibroblast ECM gene expression and contractile activity through its binding to fibroblast αvß_3_ integrin in an autocrine manner.

In our study, dermal fibroblasts cultured with TGF-ß1, a factor know to be critical to skin fibrosis following injury, had significantly increased *MFAP5* expression^61,62^. This finding agrees with previous work in which preadipocytes cultured with TGF-ß1 also had increased *MFAP5* expression^50^. Furthermore, TGF-ß1 significantly increased MFAP5 protein expression in neonatal dermal fibroblasts. Therefore, TGF-ß1 may activate a cellular pathway upstream of MFAP5 production. MFAP5 could then be a mediator of TGF-ß1, specifically TGF-ß1’s effect on cellular phenotype and transcriptome. Interestingly, it seems that MFAP5 may also bind and sequester active TGF-ß1, which further suggests it plays an active role in TGF-ß signaling in the ECM^12,13,63^. The exact relationship between MFAP5 and TGF-ß1 therefore necessitates further study.

Beyond the interaction of MFAP5 with αvß3 integrin, MFAP5 is likely to exert effects through other receptors as well. For example, MFAP5 can bind to both Notch1 receptors and ligands, which are expressed in skin fibroblasts and whose activation leads to expression of α-SMA^12,14,17,64^. In fibrotic skin disorders, including systemic sclerosis, Notch signaling may be activated and cause subsequent increased release of collagen and differentiation of fibroblasts into myofibroblasts^64^. Taken together, it seems that MFAP5 has multiple potential pathways by which it can be involved in the pathogenesis of fibrotic diseases.

In addition to changes in collagen synthesis, our in vivo results show that MFAP5 promotes wound angiogenesis. Prior studies have shown that MFAP5 can activate endothelial cell sprouting by antagonizing Notch signaling^17,52^ and promotes angiogenesis and micro-vessel leakiness through activation of lipoma-preferred partner (LPP)^19^. Further evidence that MFAP5 might regulate wound angiogenesis comes from its expression pattern in wounds. Peak MFAP5 expression in wounds was seen to occur during the stages of wound healing where angiogenesis may occur. Both basic and clinical studies have linked scar formation to increased microvascular content and an excessive angiogenic response during wound repair^7,65–68^. Our findings therefore suggest that in addition to direct effects on fibroblasts, MFAP5 may increase fibrosis via enhanced angiogenesis.

Lastly, our analysis of previously identified fibroblast subpopulations in mouse wounds indicates that specific fibroblast subpopulations are responsible for MFAP5 expression during wound healing. Based on our GO term enrichment analysis, it is likely that these fibroblast subpopulations distinctly contribute to ECM deposition and organization. This coincides with our in vivo studies suggesting that MFAP5 may be important for collagen synthesis and our in vitro studies suggesting that MFAP5 regulates expression of ECM proteins in fibroblasts. Since our work shows that MFAP5 may influence fibroblast phenotype and transcriptome in vitro, it would be interesting to study the direct effects of secreted MFAP5 on fibroblast subpopulations in which it is not differentially expressed. It is possible that certain fibroblast subpopulations may react differently to MFAP5. Overall, examining MFAP5 in the context of fibroblast heterogeneity in vivo offers another interesting facet by which we can study the role of microfibrillar proteins in wound healing.

Our study characterizes a new role for MFAP5 in wound healing. Our key findings that MFAP5 is upregulated in healing wounds and promotes fibroblast differentiation towards a pro-fibrotic phenotype support a novel role for MFAP5 in the regulation of fibroblasts, scar formation, and ECM reconstruction in skin wound healing. Importantly, neutralization of MFAP5 with an anti-MFAP5 antibody in a healing wound resulted in decreased angiogenesis and collagen deposition. This information provides a unique perspective on the potential role of this multifunctional glycoprotein in wound repair and suggests that the study of microfibrils and their associated proteins in wound healing will be a rewarding area of future research.

## Supplementary Information

Supplementary Information 1.

Supplementary Information 2.

Supplementary Information 3.

Supplementary Information 4.

Supplementary Information 5.

Supplementary Information 6.

Supplementary Information 7.

Supplementary Information 8.

Supplementary Information 9.

Supplementary Information 10.

## Data Availability

The RNA sequencing data sets will be openly available upon publication in NCBI GEO  with the accession number GSE224202. The mouse wound sc-RNA seq data analyzed in this study were a re-analysis of existing supplementary data, which are openly available at locations cited in the reference section. Raw data sets for each figure and supplementary figure are included in Supplementary Information 2 and 3. Differential expression analyses for the 3 contrasts are included in Supplementary Information 4, 6 and 7. Normalized, raw, and log2foldchange transformed count data are included in Supplementary Information 5, 8, and 10, respectively. Raw data for the ANOVA performed between all groups is included in Supplementary Information 9.
