# Supplementary material for: Microfibril-associated protein 5 and the regulation of skin scar formation
Source: Sci Rep. 2023 May 30;13:8728. doi: 10.1038/s41598-023-35558-x (PMC10229580; doi:10.1038/s41598-023-35558-x)
Supplement: Supplementary file 1 — Supplementary Information 1. [file 41598_2023_35558_MOESM1_ESM.pdf]

Supplementary Table 1. Primer sequences for real time PCR

Protein Name Abbreviation	Gene Name	Forward Primer (5'-3')	Reverse Primer (5'-3')	Species
COL1A1	*COL1A1*	TGACGAGACCAAGAACTGCC	GCACCATCATTTCCACGAGC	Human
COL3A1	*COL3A1*	AAGTCAAGGAGAAAGTGGTCG	CTCGTTCTCCATTCTTACCAGG	Human
COL6A1	*COL6A1*	GAGACGCAGTGAGTGGGAAA	CAGCCGCACCATTTTTGACA	Human
COL11A1	*COL11A1*	CAGATAGATGAGGCAAACATCG	ATACCAGGCTCAACCACTGC	Human
MMP1	*MMP1*	GCACAAATCCCTTCTACCCG	TGAACAGCCCAGTACTTATTCC	Human
MMP9	*MMP9*	CGAACTTTGACAGCGACAAG	CACTGAGGAATGATCTAAGCCG	Human
α-SMA	*ACTA2*	CCGGGACTAAGACGGGAATC	ATGGGGACATTGTGGGTGAC	Human
CTGF	*CTGF*	ACCTGTGCCTGCCATTAC	TCTCTCACTCTCTGGCTTCA	Human
TGFß-1	*TGFB1*	CAGACTCAGCCAAGACACTATT	GCTCACTTCCAGAGAGATGATT	Human
MFAP5	*MFAP5*	ATGTCGCTCTTGGGACC	TCACAGACCATTGGGTCTC	Human
GAPDH	*GAPDH*	CAGGGCTGCTTTTAACTCTGG	TGGGTGGAATCATATTGGAACA	Human
MFAP5	*Mfap5*	TCGCAGCTCTGTAGACAATATG	GAGGGATCGTTCACCAGATTAG	Mouse
COL1A1	*Col1a1*	GGT ATG CTT GAT CTG TAT CTG C	AGT CCA GTT CTT CAT TGC ATT	Mouse
COL3A1	*Col3a1*	AGC ACC TGT TTC TCC CTT T	CTG GTA TGA AAG GAC ACA GAG	Mouse
α-SMA	*Acta2*	CAG GGA GTA ATG GTT GGA AT	TCT CAA ACA TAA TCT GGG TCA	Mouse
VEGF	*Vegf*	TGC AGG CTG CTG TAA CGA TG	GAA CAA GGC TCA CAG TGA TTT TCT	Mouse
GAPDH	*Gapdh*	TCA CCA CCA TGG AGA AGG	GCT AAG CAG TTG GTG GTG CA	Mouse

Supplementary Table 2. Annotated genes to top 10 CC GO terms for sC2

Rank	GO Term	P.adj	Genes
1	Collagen-containing extracellular matrix (GO:0062023)	1.64E-37	Fbn2, Spon1, Col16a1, Sparc, Col14a1, Eln, Col11a1, Col12a1, Serpine1, Dpt, Aebp1, Ltbp3, Thbs2, Mdk, Abi3bp, Timp2, Cthrc1, Postn, Lum, Serpinf1, Mmp2, Bgn, Ssc5d, Aspn, Dcn, Col1a1, **Mfap5**, Mfap4, Smoc2, Sfrp1, Col3a1, Col1a2, Cilp, Col5a2, Ogn, Mfap2, Col8a1, Angptl4, Fmod, Angtl1, Fbn1
2	Endoplasmic reticulum lumen (GO:0005788)	1.51E-09	Spon1, Col16a1, Ifgbp4, Col14a1, Ifgbp3, Col11a1, Col12a1, Fstl1, Col1a1, Col3a1, Col1a2, Col5a2, Mxra8, Col8a1, Gas6, Fbn1
3	Intracellular organelle lumen (GO:0070013)	2.86E-09	Spon1, Col16a1, Sparc, Tnfaip6, Col14a1, Col11a1, Col12a1, Fstl1, Timp2, Igfbp4, Lum, Igfbp3, Bgn, Dcn, Col1a1, Col3a1, Col1a2, Col5a2, Ogn, Mxra8, Col8a1, Ptx3, Gas6, Fmod, Fbn1
4	Supramolecular fiber (GO:0099512)	3.20E-08	Fbn2, **Mfap5**, Mfap4, Eln, Mfap2, Fbn1
5	Microfibril (GO:0001527)	7.47E-08	Fbn2, **Mfap5**, Mfap4, Mfap2, Fbn1
6	Lysosomal lumen (GO:0043202)	2.62E-04	Lum, Gaa, Ogn, Bgn, Fmod, Dcn
7	Golgi lumen (GO:0005796)	5.30E-04	Lum, Ogn, Bgn, Gas6, Fmod, Dcn
8	Platelet alpha granule (GO:0031091)	2.99E-03	Sparc, Serpine1, Igf1, Gas6, Thbs2
9	Elastic fiber (GO:0071953)	3.93E-03	Mfap4, Eln
10	Vacuolar lumen (GO:0005775)	5.02E-03	Lum, Gaa, Ogn, Bgn, Fmod, Dcn

Supplementary Table 3. Annotated genes to top 10 BP GO terms for sC2

Rank	GO Term	P.adj	Genes
1	Extracellular matrix organization (GO:0030198)	1.32E-20	Fbn2, Col16a1, Sparc, Col14a1, Eln, Col11a1, Col12a1, Serpine1, Dpt, Timo2, Postn, Lum, Mmp2, Bgn, Dcn, Col1a1, **Mfap5**, Smoc2, Col3a1, Col1a2, Lox, Col5a2, Itga11, Mfap2, Col8a1, Gas6, Fbn1
2	Extracellular structure organization (GO:0043062)	2.83E-19	Fbn2, Postn, Col16a1, Sparc, Col14a1, Lum, Eln, Mmp2, Col11a1, Serpine1, Bgn, Dcn, Col1a1, **Mfap5**, Smoc2, Col3a1, Col1a2, Lox, Col5a2, Itga11, Mfap2, Col8a1, Fbn1
3	External encapsulating structure organization (GO:0045229)	2.83E-19	Fbn2, Postn, Col16a1, Sparc, Col14a1, Lum, Eln, Mmp2, Col11a1, Serpine1, Bgn, Dcn, Col1a1, **Mfap5**, Smoc2, Col3a1, Col1a2, Lox, Col5a2, Itga11, Mfap2, Col8a1, Fbn1
4	Collagen fibril organization (GO:0030199)	1.10E-10	Col1a1, Col3a1, Col16a1, Col1a2, Lox, Col14a1, Lum, Col11a1, Col12a1, Col5a2, Col8a1, Dpt
5	Supramolecular fiber organization (GO:0097435)	1.74E-07	Col16a1, Col14a1, Lum, Col11a1, Col12a1, Dpt, Ltbp3, Rhob, Col1a1, Mfap4, Col3a1, Marcks, Col1a2, Lox, Col5a2, Col8a1
6	Regulation of angiogenesis (GO:0045765)	1.24E-06	Smoc2, Sfrp1, Sparc, Rgcc, Serpinf1, Serpine1, Angptl4, Thbs2, Klf4, Hk2, Dcn, Rhob
7	Negative regulation of angiogenesis (GO:0016525)	1.33E-05	Sparc, Rgcc, Serpinf1, Serpine1, Thbs2, Klf4, Dcn, Klf2
8	Regulation of insulin-like growth factor receptor signaling pathway (GO:0043567)	1.51E-05	Igfbp4, Cilp, Igfbp3, Igf1, Igfbp6
9	Eye morphogenesis (GO:0048592)	1.78E-05	Fbn2, **Mfap5**, col5a2, Mfap2, Fbn1
10	Embryonic eye morphogenesis (GO:0048048)	5.84E-05	Fbn2, **Mfap5**, Mfap2, Fbn1

Supplementary Table 4. Annotated genes to top 10 CC GO terms for sC12

Rank	GO Term	P.adj	Genes
1	Collagen-containing extracellular matrix (GO:0062023)	2.97E-29	Fbn2, Lgals3bp, Sparc, Tnxb, Col14a1, Eln, Serpine1, Dpt, Fbln1, Prelp, Thbs1, Icam1, Lfals3, Clec3b, Adamts1, Abi3bp, Timp3, S100a10, Cthrc1, Serpinf1, Rarres2, Fn1, Pcolce, Dcn, Col1a1, **Mfap5**, Smoc2, Mfap4, Sfrp1, Vcan, Col3a1, Col1a2, Cilp, Ogn, Mgp, Mfap2, Serping1, Col8a1, Fbn1
2	Endoplasmic reticulum lumen (GO:0005788)	2.19E-11	Igfbp5, Igfbp4, Col14a1, Shisa5, Fn1, Ptgs2, Prss23, Fstl1, Thbs1, Col1a1, Adamts5, Vcan, Col3a1, Col1a2, Gpc3, Serping1, Col8a1, Timp1, Gas6, Fbn1
3	Supramolecular fiber (GO:0099512)	5.04E-09	Fbn2, **Mfap5**, Mfap4, Eln, Mfap2, Fbln1, Fbn1
4	Secretory granule lumen (GO:0034774)	5.61E-09	Lgals3bp, Sparc, Fuca1, Rarres2, Serpine1, Fn1, Igf2, Cxcl1, Thbs1, Nfkb1, Plac8, Clec3b, Pnp, Serping1, Timp3, Ptx3, Timp1, Gas6
5	Intracellular organelle lumen (GO:0070013)	1.17E-08	Sparc, Tnfaip6, Col14a1, Cxcl1, Prelp, Ptgs2, Prss23, Fstl1, Thbs1, Adamts5, Pnp, Gpc3, Timp1, Igfbp5, Igfbp4, Shisa5, Fn1, Dcn, Col1a1, Vcan, Col3a1, Col1a1, Ogn, Serping1, Col8a1, Ptx3, Gas6, Fbn1
6	Microfibril (GO:0001527)	3.70E-07	Fbn2, **Mfap5**, Mfap4, Mfap2, Fbn1
7	Platelet alpha granule lumen (GO:0031093)	1.60E-06	Sparc, Serpine1, Fn1, Igf2, Serping1, Timp1, Gas6, Thbs1
8	Platelet alpha granule (GO:0031091)	1.42E-05	Sparc, Serpine1, Fn1, Igf2, Serping1, Timp1, Gas6, Thbs1
9	Platelet dense granule lumen (GO:0031089)	6.40E-05	Lgals3bp, Clec3b, Rarres2, Timp3
10	Elastic fiber (GO:0071953)	7.40E-05	Mfap4, Eln, Fbln1

Supplementary Table 5. Annotated genes to top 10 BP GO terms for sC12

Rank	GO Term	P.adj	Genes
1	Extracellular matrix organization (GO:0030198)	4.52E-17	Fbn2, Sparc, Col14a1, Eln, Serpine1, Dpt, Thbs1, Icam1, Adamts5, Adamts1, Has1, Timp1, Fn1, Dcn, Col1a1, **Mfap5**, Smoc2, Vcan, Col3a1, Col1a2, Lox, Mfap2, Col8a1, Cd47, Gas6, Cd44, Fbn1
2	Extracellular structure organization (GO:0043062)	2.93E-16	Fbn2, Sparc, Col14a1, Eln, Serpine1, Fn1, Thbs1, Dcn, Icam1, Col1a1, **Mfap5,** Adamts5, Smoc2, Vcan, Col3a1, Col1a2, Lox, Adamts1, Mfap2, Col8a1, Cd47, Cd44, Fbn1
3	External encapsulating structure organization (GO:0045229)	2.93E-16	Fbn2, Sparc, Col14a1, Eln, Serpine1, Fn1, Thbs1, Dcn, Icam1, Col1a1, **Mfap5,** Adamts5, Smoc2, Vcan, Col3a1, Col1a2, Lox, Adamts1, Mfap2, Col8a1, Cd47, Cd44, Fbn1
4	Platelet degranulation (GO:0002576)	2.46E-07	Lgals3bp, Clec3b, Sparc, Rarres2, Serpine1, Fn1, Igf2, Serping1, Timp3, Timp1, Gas6, Thbs1
5	Regulation of angiogenesis (GO:0045765)	5.38E-06	Sparc, Serpinf1, Serpine1, Klf4, Thbs1, Hk2, Dcn, Rhob, Smoc2, Sfrp1, Rgcc, Adamts1, Cd34
6	Regulated exocytosis (GO:0045055)	1.04E-05	Lgals3bp, Clec3b, Sparc, Rarres2, Serpine1, Fn1, Igf2, Serping1, Timp3, Timp1, Gas6, Thbs1
7	Negative regulation of angiogenesis (GO:0016525)	1.09E-05	Sparc, Rgcc, Adamts1, Serpinf1, Serpine1, Klf4, Thbs1, Dcn, Klf2
8	Positive regulation of extrinsic apoptotic signaling pathway via death domain receptors (GO:1902043)	1.42E-05	Sfrp1, Timp3, Thbs1, Atf3
9	Integrin-mediated signaling pathway (GO:0007229)	3.81E-05	Col3a1, Adamts1, Fn1, Itgbl1, Fbln1, Isg15, Cd47, Plpp3
10	Positive regulation of cell differentiation (GO:0045597)	4.45E-05	Fbn2, Rarres2, Lpl, Nfkb1, Tgfbr2, Col1a1, Gja1, Zfp36, Sfrp1, Rgcc, Pdpn, Il6st, Cd34

Suppl Fig. 1

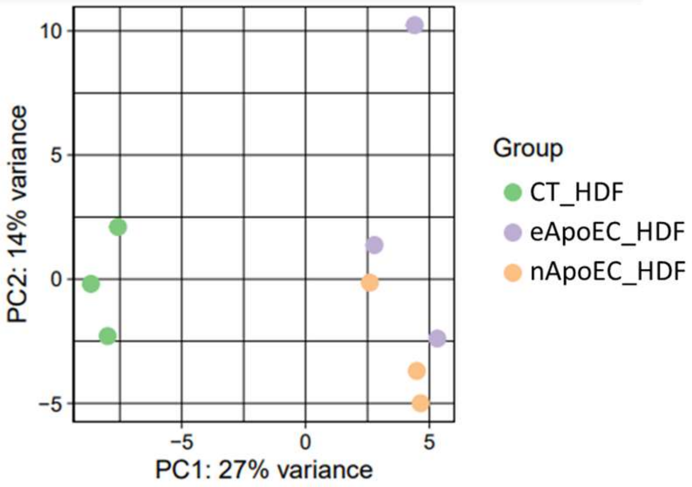

Supplementary Figure 1: Principal component analysis plot for phagocytic fibroblasts. Principal component analysis was performed and plotted for control fibroblasts (CT_HDF), fibroblasts phagocytosing apoptotic endothelial cells (eApoEC_HDF), and non-phagocytosing fibroblasts exposed to apoptotic endothelial cells (nApoEC_HDF). N=3 for each group.

Suppl Fig. 2

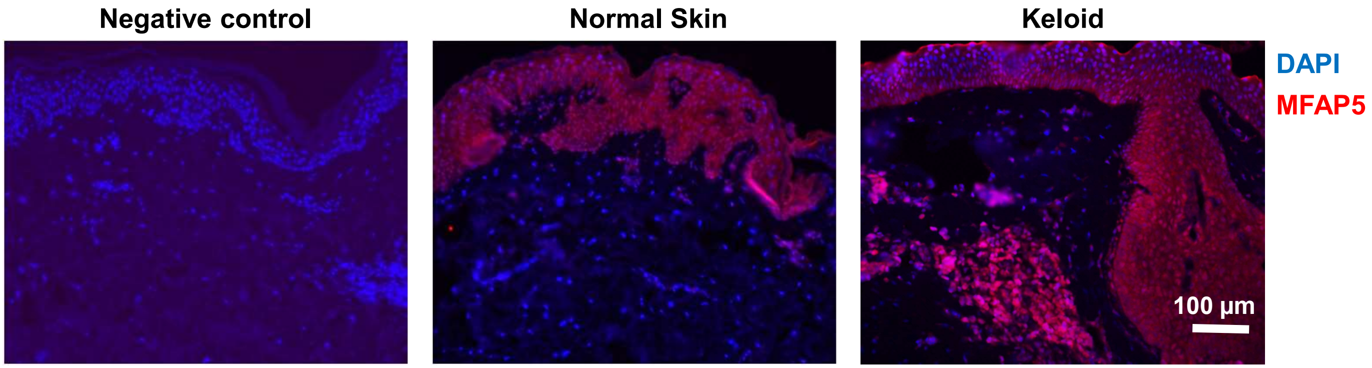

Supplementary Figure 2: MFAP5 is strongly expressed in the epidermis and dermis of human keloid tissue, but mainly in the epidermis of normal skin. A representative image of immunofluorescence detection of MFAP5 (red) in a human keloid scar (n=3) and normal skin (n=1). Nuclei in blue (DAPI).

Suppl Fig. 3

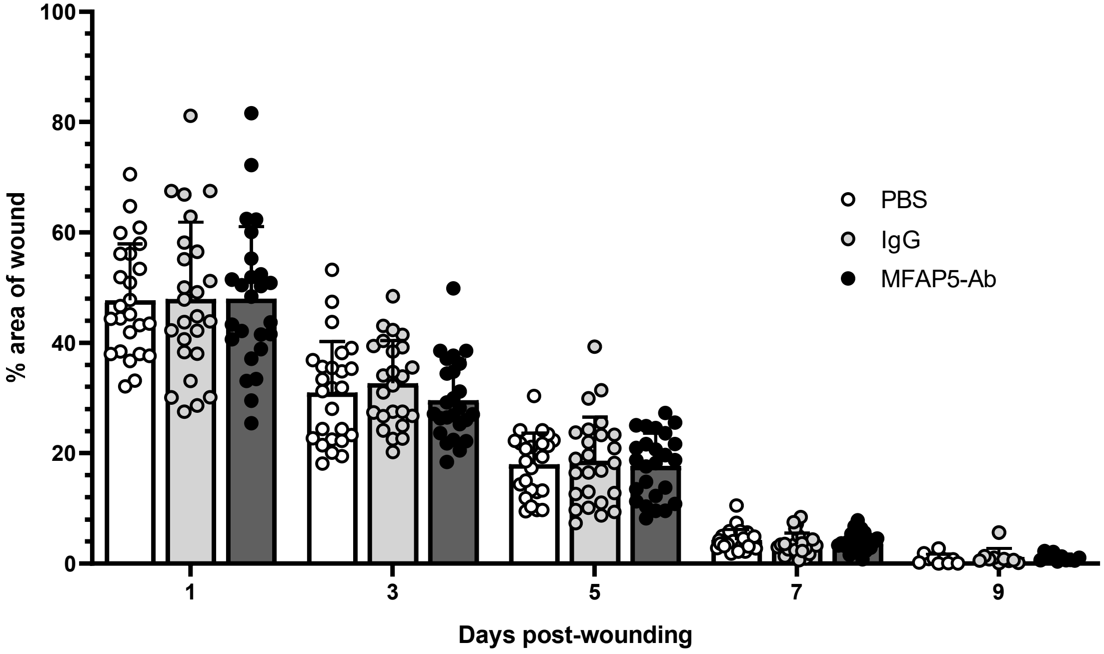

Supplementary Figure 3: Anti-MFAP5 treatment does not impair wound closure *in vivo*. Rate of wound closure in mouse wounds treated with PBS, mouse IgG or anti-MFAP5 antibody expressed as a percent of the original wound. Bars indicate mean ± SD. N=10-25 for each group; all mice were females. Two-Way ANOVA with two-stage linear step-up procedure of Benjamini, Krieger and Yekutieli (vs PBS or IgG).

Suppl Fig. 4

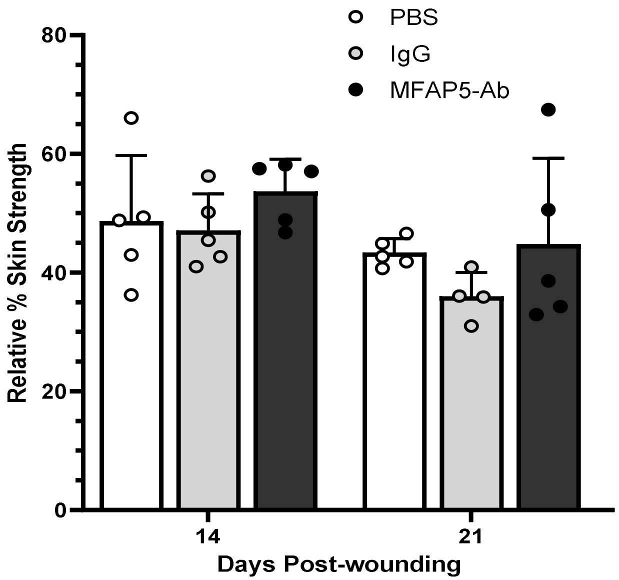

Supplementary Figure 4: Anti-MFAP5 treatment does not affect wound breaking strength *in vivo*. Relative breaking strength of mouse wounds treated with PBS, mouse IgG or anti-MFAP5 antibody as compared to normal skin. Bars indicate mean ± SD. N=4-5 for each group; all mice were females. Two-Way ANOVA with two-stage linear step-up procedure of Benjamini, Krieger and Yekutieli (vs PBS or IgG).

Suppl Fig. 5

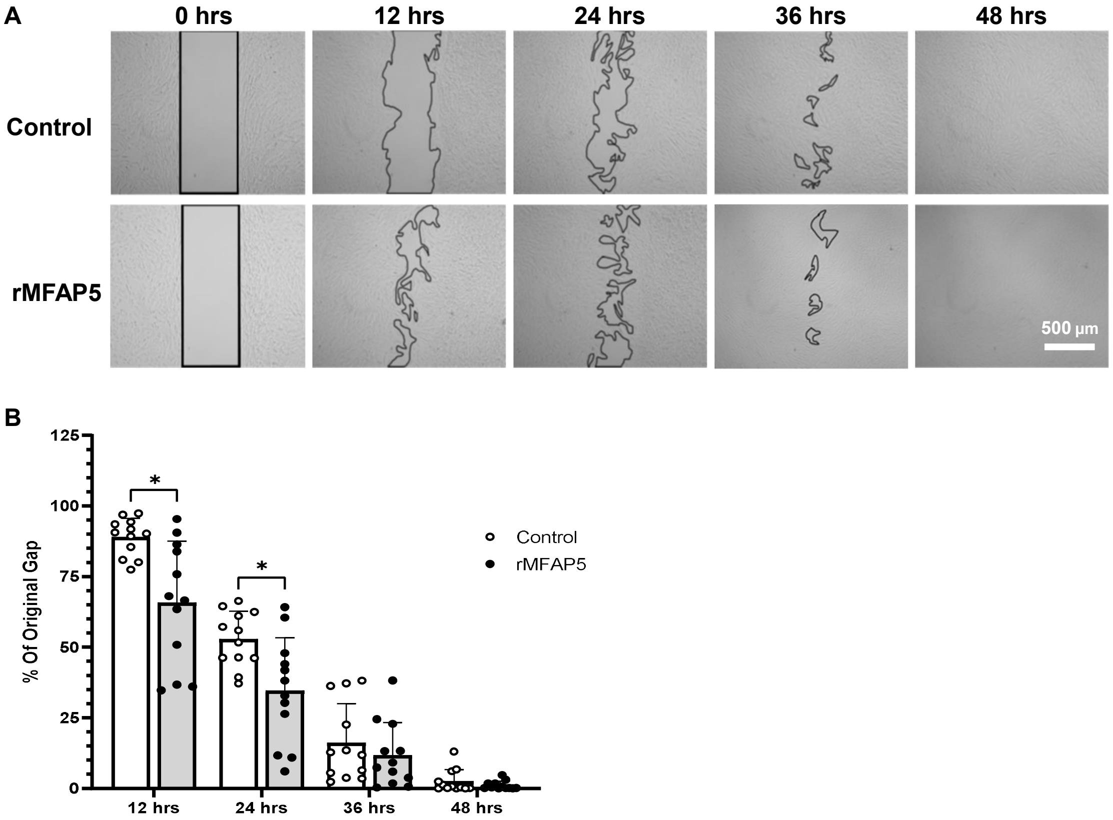

Supplementary Figure 5: Exogenous MFAP5 treatment increases fibroblast migration *in vitro* under low serum conditions. A) Representative photos of the fibroblast cell migration assay in low serum media supplemented with or without 200 ng/mL recombinant MFAP5 (rMFAP5). The areas not covered by cells are outlined by a black line. B) The rate of cell migration expressed as a percentage of the original uncovered area. Bars indicate mean ± SD. N=12 in each group. * = $p < 0.01$. Two-way ANOVA followed by two-stage linear step-up procedure of Benjamini, Krieger and Yekutieli post-hoc testing (vs Control).

Suppl Fig. 6

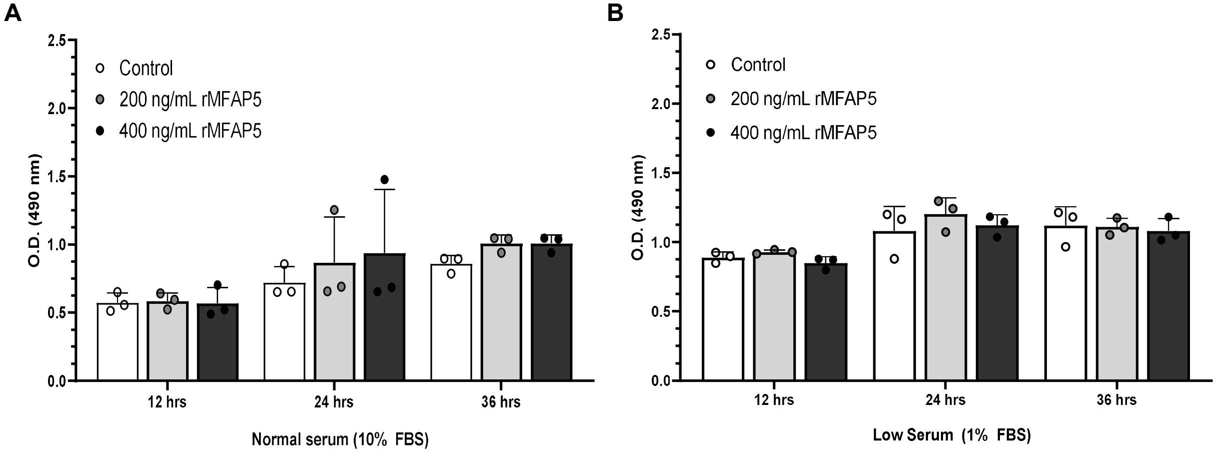

Supplementary Figure 6. Exogenous MFAP5 treatment does not affect dermal fibroblast proliferation *in vitro*. MTS proliferation assay was performed on fibroblasts treated with 200 or 400 ng/mL recombinant MFAP5 (rMFAP5) for 12, 24 or 36 hours under normal (A) or low serum (B) conditions. Bars indicate mean ± SD. N=3, with each dot representing a biological replicate that consists of 3-4 technical replicates Two-way ANOVA with two-stage linear step-up procedure of Benjamini, Krieger and Yekutieli post-hoc testing (vs Control).

Suppl Fig. 7

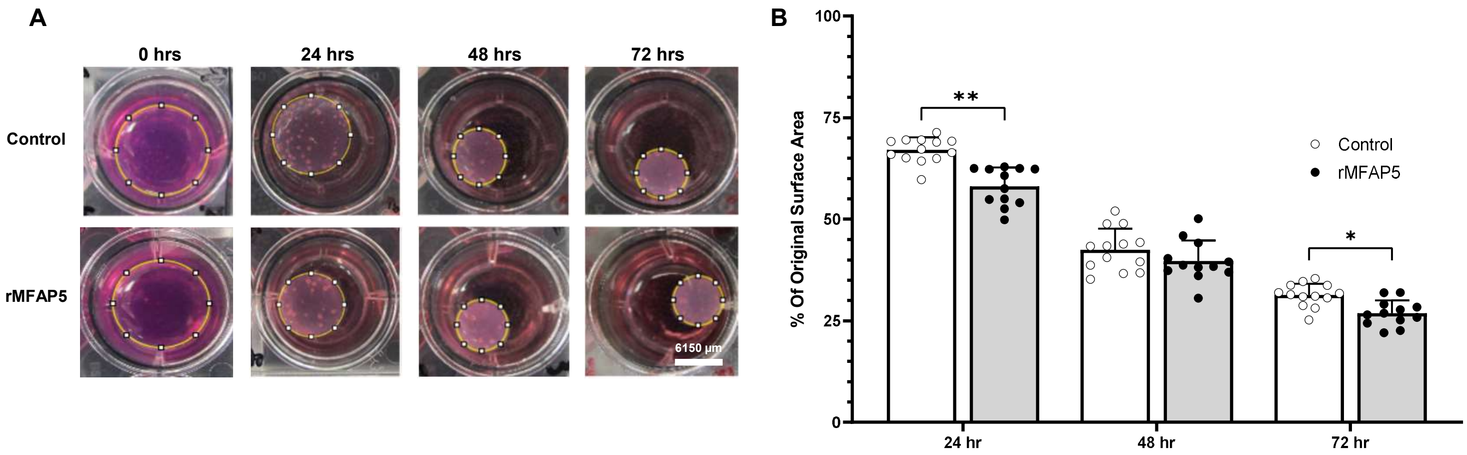

Supplementary Figure 7: Exogenous MFAP5 treatment increases collagen gel contraction *in vitro* under low serum conditions. A) Representative photos of the collagen gel contraction assay for dermal fibroblasts cultured in low serum media with or without 200 ng/mL recombinant MFAP5 (rMFAP5). The gel area is depicted by a yellow line. Scale bar = 6150 µm. B) Rate of gel contraction , expressed as a percentage of the original gel surface area. Bars indicate mean ± SD. N=13. * = $p < 0.05$, ** = $p < 0.0001$. Two-way ANOVA with two-stage linear step-up procedure of Benjamini, Krieger and Yekutieli post-hoc testing (vs Control).

Suppl Fig. 8

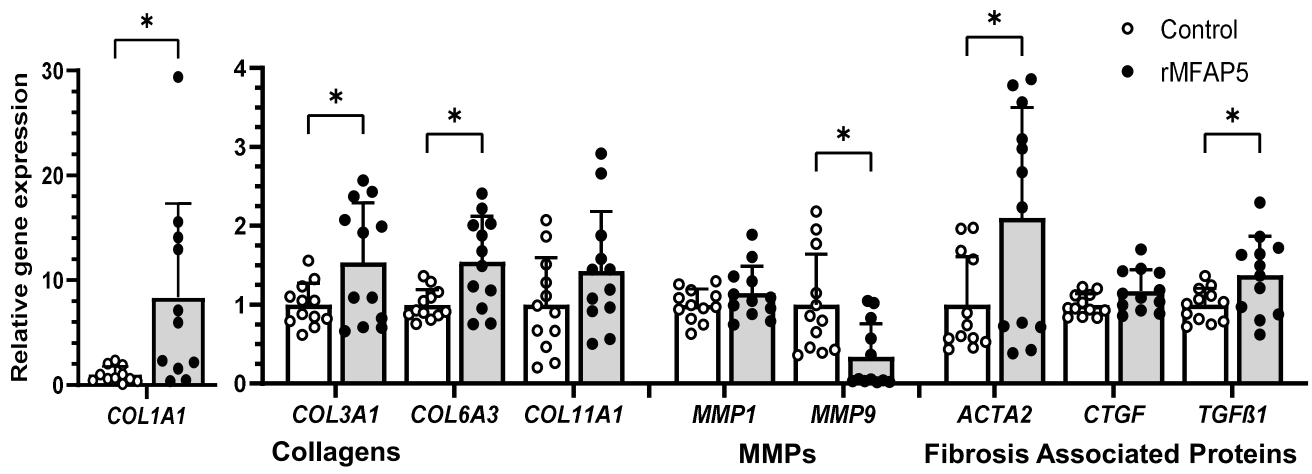

Supplementary Figure 8: Relative gene expression levels of pro-fibrotic genes and ECM proteins in dermal fibroblasts treated with exogenous MFAP5 *in vitro* under low serum conditions. RT-PCR performed on fibroblasts cultured in low serum media with or without 200 ng/mL recombinant MFAP5 (rMFAP5) for 6 hours. Gene expression was normalized to glyceraldehyde 3-phosphate dehydrogenase (*GAPDH*) expression and expressed as $2^{-\Delta\Delta CT}$. Bars indicate mean ± SD. N=12, with each dot representing a biological replicate that consists of 3 technical replicates. * = $p < 0.05$. Two-tailed unpaired t-test with Welch's correction (vs Control).
